# *Zataria multiflora* Boiss. extract improves spatial memory and learning capacity in scopolamine-induced amnesic rats

**DOI:** 10.22038/AJP.2019.13540

**Published:** 2019

**Authors:** Vahid Sheibani, Ali Mandegary, Elham Vazifekhahan, Zahra Kasbzade, Amir Asadi, Fariba Sharififar

**Affiliations:** 1 *Neuroscience Research Center, Kerman University of Medical Sciences, Kerman, Iran*; 2 *Pharmaceutical Research Center, Neuroscience Institute, Department of Toxicology, Faculty of Pharmacy, Kerman University of Medical Sciences, Kerman, Iran*; 3 *Herbal and Traditional Research Center, Department of Pharmacognosy, Faculty of Pharmacy, Kerman University of Medical Sciences, Kerman, Iran*

**Keywords:** Anticholinesterase, Cognitive disorder, Morris water maze, Passive avoidance test, Zataria multiflora

## Abstract

**Objective:**

*Zataria multiflora* (*Zm*) has been proposed for memory enhancing in Persian traditional medicine; but to now, no study has been carried out in this field yet. The aim of this research was to study the plant effect on spatial memory in scopolamine-induced amnesia and investigate *in vivo* anticholinesterase effect of *Zm*.

**Material and Methods:**

Aerial parts of the plant were extracted with methanol and standardized on the basis of rutin content. Male rats received three doses of *Zm* extract (100, 200 and 400 mg/kg, intraperitoneal (ip) for 7 days) and 30 min after the latest dose, scopolamine (1 mg/kg) was administered to animals. Learning capacity and spatial memory were studied using morris water maze (MWM) and passive avoidance test (PAT) methods. Anticholinesterase activity was studied using Ellman’s method. Physostigmine (0.3 mg/kg) and piracetam (200 mg/kg) were used as positive controls.

**Results:**

All doses of *Zm* extract significantly decreased the distance and time spent to find the platform in MWM and increased the time latency in PAT test. In both MWM and PAT tests, the highest effect of *Zm* was observed at 200 mg/kg which was in accordance with AChE inhibitory effect of the plant.

**Conclusion:**

Our findings indicate that *Zm *has anti-amnesic effect and might improve memory deficit through anticholinesterase activity.

## Introduction

One of the most recent health problems is cognitive impairment and memory deficiency that are happening very quickly and require proper preventive strategies. Memory is the ability of acquiring and processing information, and storing and retrieving them in a long and short periods of time (Mayford et al., 2012; Stuchlik, 2014). Alzheimer’s disease (AD) is a common and gradually progressive neurodegenerative disorder which affects memory, personality and behavioral functions (Khanam et al., 2016). Some of therapeutic agents such as AChE inhibitors (AChEIs) can increase the memory and are a promising approach to improve cognitive deficits. A variety of synthetic and natural sources have been studied to achieve the novel AChEIs agents (Bin Sayeed et al., 2013, 2014; Chaiyana et al., 2013; Mandegary et al., 2014b). 

*Zatraia multiflora* Boiss. (*Zm*) (Persian name “*Avishane-shirazi*”) from Lamiaceae family, is widely distributed in Afghanistan, Pakistan and throughout Iran, especially in different regions of Kerman province (Sharififar, 2011, 2014; Mohamadi et al., 2015). In Persian traditional medicine, this plant has been recommended for memory enhancing, recall increasing and mind brightness (Momen, 2009). Aerial parts of the plant have anticonvulsant, hepatoprotective, antiviral and antimutagenicity activity (Barati et al., 2010; Dehghan-Noodeh et al., 2013; Mandegary et al., 2013; Ahmadipour et al., 2015; Sharififar et al., 2015). Recent studies revealed antioxidant and *in vitro* anticholinesterase effects of the plant and separated fractions (Sharififar et al., 2007, 2011a, 2011b). In the present work, we intended to evaluate the effectiveness of *Zm *on memory impairment in scopolamine-induced amnesia using morris water maze (MWM) and passive avoidance test (PAT) and study *in vivo* anticholinesterase effect of the plant.

## Materials and Methods

**Plant materials**

Leaves and short branches of *Zm* were gathered from Kerman province in July 2016. A herbarium sample of the specimen after authentication by Dr. Mirtdazadini, Shahid Bahonar University, was deposited in the Herbarium Center of Faculty of Pharmacy, Kerman University of Medical Sciences, Kerman, Iran (KF1241). About 500 g of the dried plant was milled, further sieved (Mesh 300), and extracted with ethanol 80% using warm maceration method for 72 hr. Filtered extract was replaced with fresh solvent after 24 hr. Obtained extracts were concentrated by rotary evaporation and dried at less than 40°C in oven. Dried extract was weighed and kept at -20°C for future experiments.

**Phytochemical screening and standardization**

Phytochemical study of the plant was carried out for the presence of alkaloid, flavonoids, terpenoids, tannins and saponins (Mandegary et al., 2012). About 10 µl of *Zm* extract and various standards of flavonoids including rutin, quercetin, kampferol, apigenin and luteolin (100 µg/ml) was spotted on a silica gel G60 F_254_ plate (Merck, Germany) and developed in mobile phase of ethyl acetate: water: formic acid: glacial acetic acid (100:27:11:11). After drying, the plate was observed under UV light at 254 and 365 nm and was sprayed with natural product reagent. Determination of R_f_ values and the color of spots revealed that different flavonoids are present in *Zm* extract, however, as the color of rutin-related spot appeared more intense than the others, rutin was used for standardization. A stock methanolic solution of rutin was prepared (50 µg/ml); 1 ml of this solution was mixed with an equal volume of aluminum chloride 2%, and incubated at room temperature for 30 min; next, the absorbance spectrum was provided and maximum wavelength was determined. Serial dilutions of rutin (2.5, 5, 10, 25 and 50 µg/ml) and two concentrations of the plant extract (50, and 100 µg/ml) were prepared and the absorbance was recorded as mentioned above. Calibration curve of rutin was used for determination of total flavonoid content (Ansari et al., 2011).

**Animals**

A number of 126 Wistar rats with the mean weight of 200±20 g (6-8 weeks) were used in this experiment. All injections were administered intraperitoneally between 8 and 10 am and all experiments were performed during “light” time (i.e. between 8 am and 4 pm) in order to prevent the effects of night-time rhythm of the animal on the experiments. Animals were adapted to laboratory conditions for one hour before the test. In all groups, the injection volume was 1 ml/kg, and the doses of *Zm* were selected based on preliminary studies (Mandegary et al., 2013). The rats were kept in individual cages at standard conditions (12 hr light, 12 hr darkness, at 23°C) and allowed to have free access to water and food. Protocol of the work with animals was in accordance with NIH guide and approved by Ethics Committee of Research Center of Kerman University (EC/KNRC/94-17). 

**Experimental animals**

**Figure 1:**
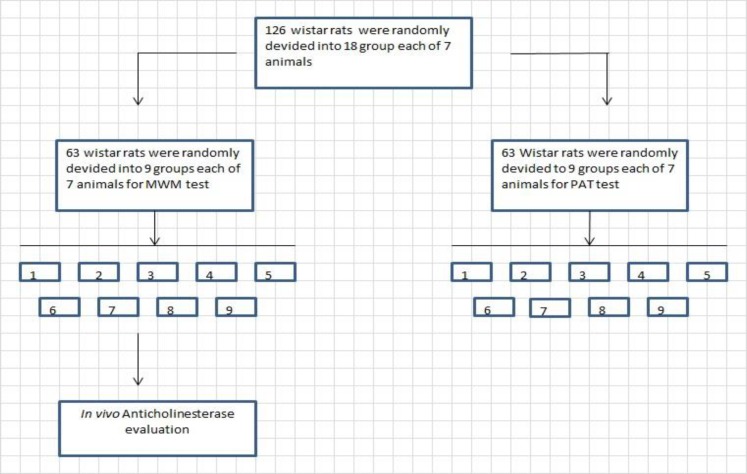
Flowchart of experimental groups of animals used for behavioral tests

The rats were randomly divided into 18 groups of 7 animals; thus, in MWM and PAT test, nine separate groups were considered (Figure 1). Scopolamine (Merck, Germany) was administered at the beginning of training phase in both MWM and PAT experiments (Table 1).

**Table 1:** Animals grouping and treatments

**No.**	**Group**	**Drug/ treatment**
**1**	Control	No treatment
**2**	Sco	Scopolamine (Sco) treated (1 mg/kg*, i.p*)
**3**	NS	Normal saline (7 consecutive days), scopolamine (1 mg/kg, *i.p*) injected on the 7^th^ day, 30 min after the latest dose of normal saline
**4-6**	*Zm*	*Z. multiflora* extract at doses of 100, 200, 400 mg/kg, *i.p* respectively (once daily, 7 consecutive days), scopolamine (1 mg/kg, *i.p*) injected on the 7^th^ day, 30 min after the latest dose of extract
**7**	Solvent	Solvent of extract (7 consecutive days), scopolamine (1 mg/kg, *i.p* ) injected on the 7^th^ day, 30 min after the latest dose of solvent (DMSO 10% in NS)
**8**	Pir	Piracetam (200 mg/kg, 7 consecutive days) scopolamine (1 mg/kg, *i.p*) injected on the 7^th^ day, 30 min after the latest dose of piracetam
**9 **	Phys	Physostigmine (0.3 mg/kg, 7 consecutive days), scopolamine (1 mg/kg, *i.p*) injected on the 7^th^ day, 30 min after the latest dose of physostigmine

**Morris water maze (MWM)**

Morris water maze was designed for evaluation of spatial memory in a short time through learning trials. The experiment was carried out in a black rounded pool with 160 cm diameter and 60 cm height and full of water 24±2°C. The pool was divided into four equal parts. A square platform (10*10 cm) was placed below the water in the center of one quadrant named target quadrant (Q4). Training period contained 3 blocks which were taken at 30 min intervals. In each block, the animal performed four successive trials from four different quadrants (north, south, east or west of the pool). In these trials, the animal was released into the pool and had the opportunity to find the hidden platform within 60 sec and rest on it for 20 sec and then, return to the cage for 30 sec until the next trial. The performance of animals was observed using a camera connected to a software and computer system (Noldus Ethovision, system 7.1, Netherland). The animals who did not find the platform after 60 sec, were guided to it. The time taken and the distance traveled to find the platform were recorded and analyzed. Two hours after training phase, the single probe test was performed for spatial memory evaluation. In the probe test, which was conducted to study the effectiveness of the extract on memory recall, the platform was removed and animals were released into water from the quadrant opposite to Q4 to freely swim for 60 sec. The number of entries to Q4, and the time spent and the distance traveled to Q4 were recorded. The last test of visible platform was conducted 2 min after completing the probe test. In visible platform test, the platform was placed about 2 cm above the water level in the quadrant opposite to Q4 and animals were released into the water to find the platform. The time taken and distance moved were recorded and analyzed (Harandi et al., 2015). 

**Passive avoidance test (PAT)**

In passive avoidance test (PAT), a shuttle box composed of a Plexiglas box was used; the apparatus had two equal dark and light compartment (20×20×20 cm) separated by a remote door. The dark part was covered by a black roof, with steel rod in the bottom arranged at a distance of 1 cm, connected by a cable to a device which generates a high intensity shock (0.5 mA, 50 Hz, 2 sec). When the animal enters the light partition, due to the intrinsic tendency of animal to darkness, it immediately moves away into the dark part; in other words, a non-active avoidance was formed. To study the long-term memory in this method, the training and the trial phases were carried out with a 24-hr interval. In the training stage, the animal slowly entered the light side, after 10 sec, the door was opened and the animals were allowed to enter the dark side and time latency was recorded. The animals returned to the cage and if the time latency was more than 100 sec, the animal was removed from the experiment. After 30 min, the animal was transferred to the light part, the remote door was opened after 10 sec and permitted animal to enter the dark side. The door was closed and an electric shock was given to the animal’s hands and feet. After 20 sec, the animal was moved to its cage. The animals that did not arrive at the dark partition after 120 sec, were removed from the experiment. Trial phase was carried out 24 hr later to study the memory of animal. No electric shock was given at this stage, but the trained animal was transferred to the light. After 10 sec, the remote door was opened, and the time latency to arrive and the number of entrance to the dark part were recorded. The test was terminated after 300 sec (Garcia-Pardo et al., 2017).

***In vivo*** **anticholinesterase activity**

In *in vivo* studies, the same animals which had been used in the behavioral experiment (in this experiment: MWM test) were decapitated swiftly using guillotine after 1 hr. The whole brain of the animal was removed, placed on ice and washed with normal saline (NS). Hippocampus was isolated and homogenized in the lysis buffer (at 9500 rpm). Anticholinesterase activity was determined by Ellman’s method. In this test, thiocholine resulted from the hydrolysis of thiocholine acetate by AChE, reacts with dithionitrobenzoic acid (DTNB) and the resulted 5-thio-2-nitrobenzoic acid anion has strong absorbance at 412 nm. Tris buffer was mixed with DTNB (50 µl, 0.01 M in Na buffer, 0.1 M, pH 8), and the absorbance was read at 412 nm using a microplate reader (Bioteck, America). After addition of acetylthiocholine iodide (ATCI) (200 µl, 75 mM) and buffer containing hippocampus (100 µl) and incubation at room temperature, the absorbance was measured at 412 nm (with 30 sec intervals up to 5 min). The same mixture without hippocampus, was used as a blank. Enzyme activity was calculated as follows:

AChE specific activity = (ΔA412×Vol T×1000)/(1.36×104×Light path×Vol S) where ΔA 412 is the changes per min at 412 nm, Vol T is the total volume of DTNB+ sample+ATCI, 1.36×104 is the molar extinction coefficient of TNB-, light path is 1 cm and Vol S is the volume of sample (Mandegary et al., 2014a). 

**Statistical analysis**

The results are presented as Mean±SEM. The results were analyzed using SPSS version 16.0. One way ANOVA and Tukey *post hoc* were used for comparison of data obtained in MWM and PAT. Velocity and latency in MWM was analyzed by repeated measure ANOVA. In all experiments, differences with p<0.05 were considered statistically significant.

## Results

**Extraction and standardization**

**Figure 2:**
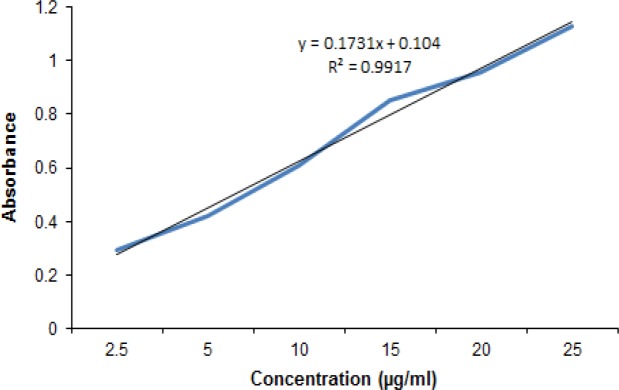
Calibration curve of rutin at maximum wavelength of absorption (ʎmax: 410 nm)

The yield of total extraction was about 17.01% w/w (extract/ dried plant). Presence of flavonoids, saponins, tannins and terpenoids was confirmed in the plant. Presence of flavonoids was shown by PEW and Pb acetate tests, terpenoids of the plant formed red-brown color with sulforic acid in Liberman–Burchard test, presence of saponins was confirmed by the froth test and tannins of the plant showed a deep blue color with ferric chloride reagent. Total flavonoid content of the *Zm* extract was estimated to be about 44.20 mg rutin equivalent flavonoid/1 g dried extract on the basis of rutin calibration curve (Figure 2). 

**MWM result**

The results of MWM test were evaluated in two discriminate trials:

Training trial: results obtained in this experiment suggested that scopolamine (Sco) caused a significant increase in both swimming distance and time latency in comparison to NS which indicate a disturbance in learning (p<0.001). No significant difference was found among animals receiving Sco, NS or solvent in three tested blocks. ANOVA analysis indicated that different doses of *Zm* extract, physostigmine (Phys) and piracetam (Pir) significantly reduced the distance moved through the block 2 and 3 (p<0.001) but not in block 1 (p>0.005). The results also showed that the time latency in all three blocks in experimental groups, has significantly decreased compared to NS (p<0.05 and p<0.001) (Table 2). 

**Table 2:** Effect of different doses of *Z. multiflora* extract on the time latency and distance moved in training trial through block 1-3 as assessed by morris water maze (MWM) test

Experimental group	Time latency (sec)			Distance moved (cm)		
	Block 1	Block 2	Block 3	Block 1	Block 2	Block 3
NS	59.72±0.16	51.73±1.89	42.45±1.29	1074.97±28.0	970.48±13.44	835.07±15.39
Solvent	56.37±1.74	47.51±2.29	39.25±1.51	1007.76 ±35.64	905.30±21.42	766.37±26.22
Sco	59.07±0.74	50.03±2.74	41.23±1.02	1155.68±31.33	989.89±33.43	822.30±32.93
Control	43.84±6.47^***^	26.33±6.17^***^	20.04±2.73^***^	721.12±23.80^***^	568.31±19.40^***^	353.28±54.49^***^
Pir	46.15±3.95^**^	36.98±3.72^**^	22.03±5.07^***^	1028.25±44.71	727.08±36.3^***^	428.07±27.11^***^
Phys	41.09±1.76^***^	25.73±1.22^***^	16.79±0.82^***^	960.17±56.84	627.90±33.33^***^	310.15±24.62^***^
*Zm* 100	41.32±4.26^***^	30.02±4.48^***^	17.99±3.6^***^	909.41±14.97	532.74±44.65^***^	283.80±52.35^***^
*Zm* 200	38.32±3.44^***^	25.79±3.21^***^	13.89±0.62^***^	904.71±23.63	415.74±34.9^***^	237.45±13.9^***^
*Zm* 400	41.41±0.87^***^	27.31±3.08^***^	16.74±4.59^***^	930.75±48.68	575.79±19.33^***^	281.66±28.02^***^

The experimental groups were treated with piracetam (Pir, 200 mg/kg), Physostigmine (Phys, 0.3 mg/kg) and *Z. mutiflora* extract (Zm, 100, 200 and 400 mg/kg) for 7 consecutive days. Animals received 1 mg/kg scopolamine (Sco) (i.p), 30 minutes after the last dose. The time latency and distance moved for finding hidden platform were recorded and compared to data from normal saline (NS), Solvent and Sco groups as assessed by MWM test in training trial. Each group contained 7 animals and the results were reported as mean±SEM. ^*** ^and^ ** ^significantly different from normal saline at p<0.001 and p<0.05, respectively.

**Figure 3:**
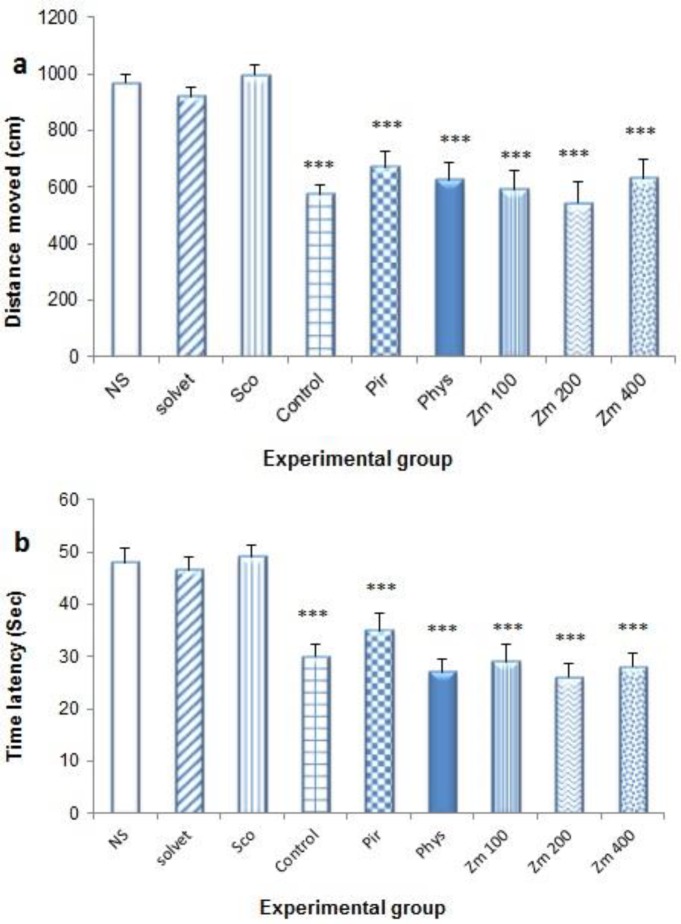
Effect of different doses of *Z. multiflora* extract on memory deficiency as assessed by morris water maze (MWM) experiment (probe phase). Animals (each of 7 rats) received the extract, drug or normal saline for 7 consecutive days. 1 mg/kg scopolamine (i.p) was administered 30 minutes after the last dose. Distance moved (a) and time latency in target quadrant (b) to find the hidden platform were recordedand data were compared to normal saline (NS), control and scopolamine (Sco) groups. The results were reported as mean±SEM. ^***^ and ^** ^significantly different from normal saline at p<0.001 and p<0.05, respectively

In probe test, the animals treated with *Zm* extract, showed significant reductions in the time latency (29.78±3.22, 26.00±2.69 and 28.49±2.66 sec at doses of 100, 200 and 400 mg/kg of *Zm*, respectively and 48.50±2.4 sec in NS group) (p<0.001 for all cases). Phys (0.3 mg/kg) and Pir (200 mg/kg) reduced escape latency to 27.87±2.36 and 35.05±3.24 sec, respectively (p<0.001). Different does of 100, 200 and 400 mg/kg of *Zm* significantly decreased the distance moved from 967.35±32.64 to 592.46±67.02, 543.39±75.77 and 632.13±67.25 cm, respectively (p<0.001) (Figures 3a and b).

**Figure 4:**
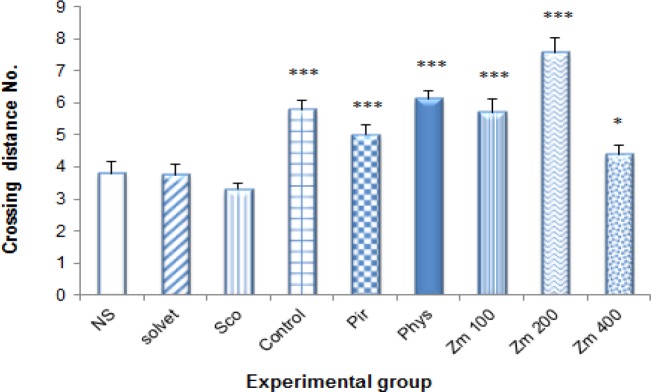
Effect of different doses of *Z. multiflora* extract on the distance crossing to target quadrant (Q4) as assessed by morris water maze (MWM) test. Different experimental groups were treated with solvent, piracetam (Pir, 200 mg/kg), physostigmine (Phys, 0.3 mg/kg) and *Z. mutiflora* extract (*Zm*, 100, 200 and 400 mg/kg) or an equal volume of normal saline (NS) for 7 consecutive days. Animals received 1 mg/kg scopolamine (i.p) 30 minutes after the last dose of drug. The distance crossing to target quadrant (Q4) was determined by morris water maze (MWM) test (probe trial) and data were compared to normal saline (NS), control and scopolamine (Sco) groups. Each group contained 7 animals and the results are reported as mean±SEM. ^***^ and ^**^ significantly different from normal saline at p<0.001 and p<0.05, respectively

These results proved that *Zm*, Pir and Phys increase the learning ability of the animal to find the platform over the whole trial, contrary to Sco which impaired learning. In this trial, Sco reduced the time spent on the goal quadrant (Q4) in comparison to NS (p<0.001). There was no significant difference among NS, control and solvent regarding the time spent, distance traveled and distance crossing in Q4. Phys increased both the time taken (53.57±3.73 seconds) and the distance traveled (60.74±2.90 cm) in Q4 compared to NS (p<0.05). Velocity did not show significant differences between experimental groups and NS (p>0.05). In this trial, distance crossing was significantly increased by Phys, Pir and *Zm* at 100 and 200 mg/kg (6.14±0.26, 5.00±0.31, 5.71±0.42 and 7.57±0.48 cm, respectively) compared to NS (3.43±0.37 cm) (p<0.05 and p<0.001) (Figure 4). 

**PAT test**

**Figure 5:**
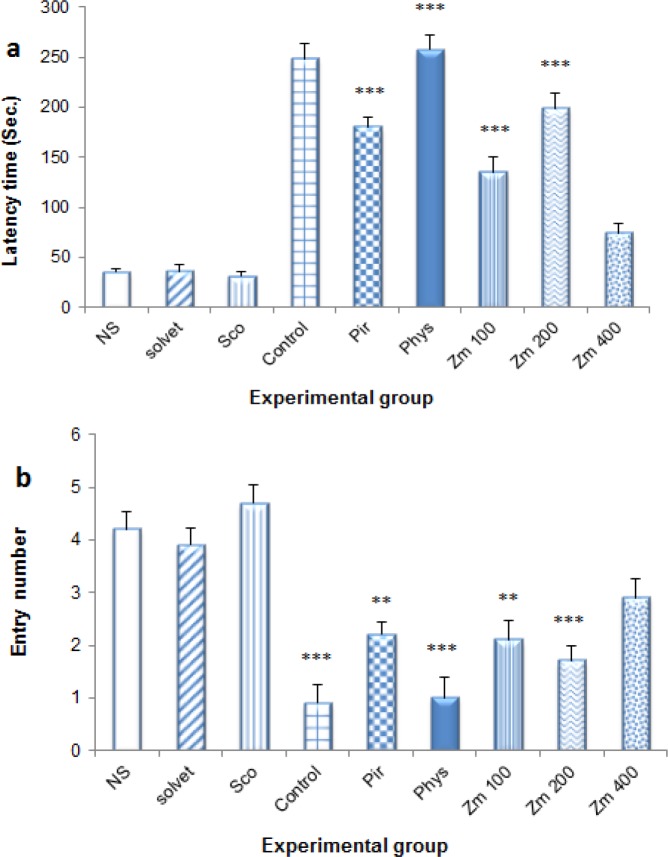
Effect of different doses of *Z. multiflora* extract on memory deficiency as assessed by passive avoidance test. Different experimental groups were treated with solvent, piracetam (Pir, 200 mg/kg), physostigmine (Phys, 0.3 mg/kg) and *Z. mutiflora* extract (Zm, 100, 200 and 400 mg/kg) or an equal volume of normal saline (NS) for 7 consecutive days. Animals received 1 mg/kg scopolamine (i.p) 30 minutes after the last dose. The time latency (a) and distance moved (b) to the dark side were determined by passive avoidance test (PAT) and data were compared to normal saline (NS), control and scopolamine (Sco) groups. Each group contained 7 animals and the results are reported as mean±SEM. ^***^ and ^** ^significantly different from normal saline at p<0.001 and p<0.05, respectively

The results of PAT test showed that Sco significantly reduced the time delay to enter to the dark part in comparison to control group (31.13±22.3 and 248.28±32.92 sec, respectively) (p<0.001), contrary to Phys and *Zm* (200 mg/kg) which significantly increased the time latency (257.84±42.93 and 198.79±19.67 seconds in comparison to NS (35.16±13.22 seconds) (p<0.001 and p<0.05 respectively). The other experimental groups induced no significant effect (p>0.05). Sco also increased the entry frequency to the dark part in comparison to control while *Zm* (200 mg/kg) and Phys significantly decreased it in Sco treated animals (1.71±0.28 and 1.00±0.38 with p<0.05 and p<0.001 respectively). The other groups were not significantly different from NS (p>0.05) (Figure 5a, b).

**Anticholinesterase activity**

**Figure 6:**
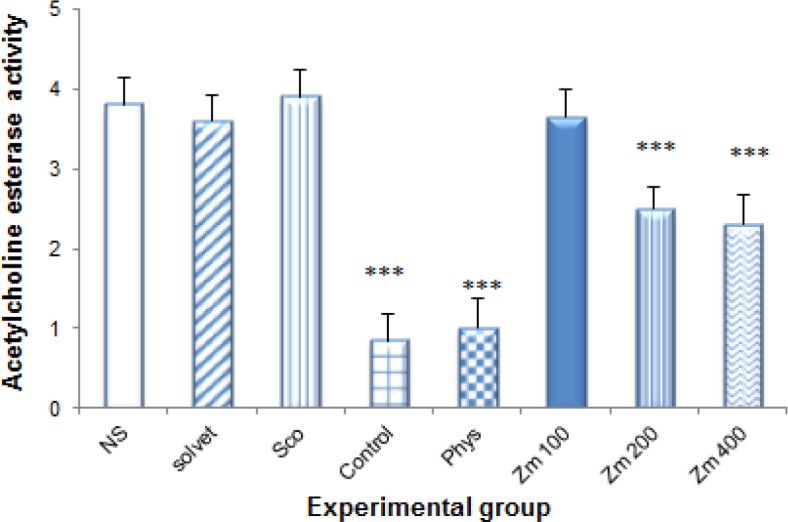
*In vivo* acetylcholinestearse inhibitory effect of different doses of *Z. multiflora* extract. Different experimental groups were treated with solvent, physostigmine (Phys, 0.3 mg/kg) and *Z. mutiflora* extract (*Zm*, 100, 200 and 400 mg/kg) for 7 consecutive days. Animals received 1 mg/kg scopolamine (i.p) 30 minutes after the last dose. Acetylcholinesterase specific activity was determined by Ellman’s method and data were compared to normal saline (NS), control and Sco groups. Each group contained 7 animals and the results are reported as mean±SEM. ^***^ and ^**^ significantly different from normal saline at p<0.001 and p<0.05

Multiple dose injection of *Zm* extract could inhibit AChE in the brain hippocampus of Sco–induced amnesic rats in a dose-dependent manner (Figure 6). Maximum inhibition was shown by Phys which decreased enzyme activity to 0.85±0.09 and *Zm* at 200 and 400 mg/kg which reduced AChE activity to 2.36±0.11 and 2.00±0.14, respectively in comparison to NS (3.82±0.34; p<0.001). Physostigmine significantly inhibited AChE in comparison to *Zm* (200 and 400 mg/kg) (p<0.001).

## Discussion

Impairment of memory, poor ability of learning and a decrease in recall function are features of learning and memory dysfunction in cognitive disorders. In this work, effect of *Zm* extract was studied on spatial memory in Sco-induced amnesia using MWM and PAT tests. MWM experiment can detect the spatial memory capacity of animals and PAT test has been used frequently for evaluation of memory task. In MWM, *Zm* extract caused a significant decrease in the time latency and distance moved to find the hidden platform (Figures 3a and b) and in PAT experiment, reversed the memory deficiency prompted by Sco and increased the time latency to the dark side. These effects might be due to different plant phytoconstituents such as flavonoids and terpenoids that could increase spatial learning ability (Li et al., 2014; Qin et al., 2015; Guo et al., 2016). Also, *Zm* extract inhibited AChE activity in a dose-dependent manner (Figure 6). Acetylcholine is a key neurotransmitter in intraneurons electrical conduction, and ACh activity deficit leads to cognition dysfunction (Kalaycioglu et al., 2017). Most of magnocellular of the basal forebrain are complicated in the memory task and learning. These cellular complex have almost homogenous nucleus, so that more of its neurons (90%) are cholinergic (Freddi et al., 2009). Our results indicated that *Zm* can improve memory function which is partly due to its anticholiesterase activity. Amyloid-beta (Aβ) peptides accumulation causes micro vascular inflammation which affects neurons and under normal conditions, are involved in inflammatory pathogenesis of AD (Giulian et al., 1996). Rosmarinic acid, an antioxidant and anti-inflammatory phenolic acid of *Zm *extract (Arabzadeh et al., 2013), can prevent aggregation of amyloid peptides and delays AD progression in animals. NMR characterization indicated that rosmarinic acid is able to interact with Aβ1-42 oligomer and might generate anti amyloidogenic molecules for specific targeting (Airoldi et al., 2013). Pretreatment with rosmarinic acid ameliorated memory deficit induced by Aβ via attenuation of oxidative process (Alkam et al., 2007) and inhibited AChE (Gulcin et al., 2016). We previously reported *in vivo* antioxidant effect of *Zm* (Sharififar et al., 2011a). Antioxidants can alleviate memory deficit related to AD in rat and delay its clinical development in humans. The brain and CNS contain a high amount of unsaturated lipids; so are more prone to oxidative stress compared to other body organs. Due to high oxygen consumption and lower activity of antioxidant enzymes, these tissues are involved in oxidation process through brain normal aging. Even though the accurate mechanism of *Zm* to improve spatial memory was beyond of this study, based on earlier findings, we can suggest that at least a part of the neuroprotective effects of this plant might be somewhat due to its antioxidant activity, though this still requires further studies. 

In conclusion, the results of the present work supported the improving effect of *Zm* on memory deficiency and learning insufficiency in Sco-induced amnesic animals. Different phytochemicals of the plant can promote this process through various mechanisms like anticholinesterase, antioxidant and /or anti- inflammatory effects. Safety study and clinical trials are required for more documentation.
